# Evaluation of the psychometric properties of PainChek in older general hospital patients with dementia

**DOI:** 10.1093/ageing/afaf027

**Published:** 2025-02-19

**Authors:** Elizabeth L Sampson, Nathan Davies, Victoria Vickerstaff

**Affiliations:** Queen Mary University of London—Centre for Psychiatry and Mental Health, Wolfson Institute of Population Health, London, UK; Whipps Cross Hospital, Barts Health NHS Trust—Academic Centre for Healthy Ageing (ACHA), London, UK; Queen Mary University of London—Centre for Psychiatry and Mental Health, Wolfson Institute of Population Health, London, UK; Queen Mary University of London—Barts Clinical Trials Unit, Wolfson Institute of Population Health, London, UK

**Keywords:** dementia, pain, hospitals, point-of-care systems, psychometrics, older people

## Abstract

**Background:**

Pain is common in people with dementia in general hospitals. This can be difficult to identify.

**Objectives:**

To evaluate the psychometric properties of PainChek electronic pain assessment tool.

**Design:**

Cross-sectional psychometric study.

**Setting:**

Six medical care of older people wards from two general hospitals in greater London, UK.

**Subjects:**

63 people with clinical diagnosis of dementia: mean 84 years (SD 6.7), 59% female, 69% living in their own homes, 64% white British, 77% moderate/severe dementia.

**Method:**

Psychometric evaluation of PainChek, a point-of-care electronic pain assessment tool combining artificial intelligence, facial analysis and smartphone technology. From a total of 216 assessments, we tested PainChek’s inter-rater reliability (IRR) (Cohen’s kappa), internal consistency (Cronbach’s alpha) and concurrent validity (Pearson’s coefficient) between PainChek and Pain Assessment in Advanced Dementia (PAINAD) scores at rest and post-movement [95% confidence interval (95% CI) where appropriate]. We assessed convergent validity with Symptom Management–End of Life in Dementia scale (SM-EOLD) (Pearson’s coefficient) and discriminant validity (rest vs post-movement).

**Results:**

IRR was 0.714 (95% CI 0.562 to 0.81) (rest) and 0.817 (95% CI 0.692 to 0.894) (post-movement). Internal consistency was 0.755 (rest) and 0.833 (post-movement). Concurrent validity with PAINAD was 0.528 (95% CI 0.317 to 0.690) (rest) and 0.787 (0.604 to 0.891) (post-movement). Convergent validity with SM-EOLD was −0.555 (95% CI −0.726 to −0.318) (rest) and −0.5644 (95% CI −0.733 to −0.331) (post-movement). Discriminant validity was significant.

**Conclusions:**

PainChek is a valid and reliable pain assessment tool for people with dementia in general hospitals. Further consideration will be needed for implementation into this setting.

## Key Points

- Pain is common in people with dementia in general hospitals, who may have difficulties indicating they are in pain.
- PainChek is an electronic pain assessment tool using artificial intelligence, facial analysis and smartphone technology.
- PainChek had good reliability and validity in people with dementia who were admitted to the general hospital.
- Further work is needed to explore how this tool could be integrated into workflow and existing IT systems in this setting.

## Introduction

Up to 42% of general hospital in-patients have dementia [1–3]; of these, nearly half have moderate or severe cognitive and functional impairment [1, 2]. Pain affects up to 57% of people with dementia with an unplanned medical admission [4]. Self-report is the gold standard for pain assessment [5], but people with dementia may find this hard, with difficulties communicating they are in pain [6], which is often under-detected, under-reported and poorly managed [7]. General hospital wards are a challenging environment. Staff may not know the person with dementia’s pain cues [8]. Difficulties may be exacerbated by acute illness, e.g. delirium. Poorly managed pain can lead to distressed behaviours such as rejection of care, agitation or aggression [9], which occur in 75% of people with dementia in general hospitals [10].

The American Geriatrics Association defines key domains of observational pain tools for people with communication difficulties: facial expression, vocalisation and verbalisation, body movements, changes in interpersonal interactions, changes in activity patterns or routines, and mental status changes [11]. Many tools include these domains [12, 13], but pain measurement remains subjective [14, 15]. Facial expressions are a key nonverbal pain indicator, may be more reliable in determining whether someone is in pain [16], are relatively consistent across a range of painful conditions and can be distinguished from other negative emotions [17]. However, these are still open to interpretation.

Facial action units (AUs) are the smallest building blocks of facial expressions. The Facial Activity Coding System (FACS) is an anatomical catalogue giving each individual facial action unit a unique label and description. Pain-relevant action units have been defined in experimental and clinical studies and include tightening eyelids, nose wrinkling and lips parting [18]. FACS is objective and unaffected by whether or not a person has dementia [18]. Digital facial expression decoding tools identify these sometimes-subtle changes. A point-of-care smart device application, the electronic pain assessment tool, subsequently commercialised as PainChek, combines artificial intelligence (AI), manual coding of pain signs such as vocalisations, automated facial analysis and smartphone technology [19]. Automated recognition of facial pain signs may reduce observer bias and overcome barriers to good pain management in hospitals [15]. PainChek has been validated in care home residents showing good psychometric properties [19–23] but not in general hospitals where the context is very different. For example, five manually coded PainChek items require knowledge of the person’s behaviours over the prior week, and this information may not be available to hospital staff.

Our aim was to explore the psychometric properties of the PainChek electronic pain assessment tool in hospital wards for older people. Specific study objectives were to evaluate the

1. Reliability of PainChek,
2. Validity of PainChek,
3. The reliability and validity of PainChek in an acute environment when manually coded items that require a week of prior knowledge of the person’s behaviour are removed.

## Methods

Data are derived from a cross-sectional study examining pain and discomfort in general hospital in-patients with dementia [24]. We describe methods for this psychometric validation study.

### Setting and participants

Wards from two acute general hospitals in greater London, UK, were selected for differing Care Quality Commission ratings (UK standards for clinical care) and level of implementation of dementia care programmes, serving sociodemographic diversity. We focussed on medical care of older people wards (six in total) where dementia and pain are common [25]. Clinical staff identified and approached patients who met the inclusion criteria and asked them if they would like to participate:

- Aged ≥70 years with an unplanned medical admission,
- Able to give written informed consent or, if they lacked capacity to consent to participate in a research project, an available personal consultee (next-of-kin, carer) or ‘professional consultee’,
- Documented dementia diagnosis (any cause) in clinical notes,
- We excluded patients where there were clinical concerns, i.e. imminently dying.

### Ethical considerations and consent processes

Consent procedures complied with mental capacity legislation (Mental Capacity Act 2005). If a patient agreed to participate, trained research staff conducted a structured assessment of capacity to consent. If they had capacity, written informed consent was obtained. If they did not have capacity, clinical staff identified their next-of-kin or carer. If this personal consultee agreed, they were provided with a proxy agreement form, face-to-face when visiting or via post. If clinical staff could not identify a personal consultee, we used a professional consultee (a senior member of the clinical care team not directly involved in the patient’s care). Health Research Authority and ethical permissions were granted by London Queen Square ethics committee (reference 19/LO/0036) (12 March 2019).

### General data collection process

Researchers introduced themselves to the participant and explained they were observing them. They had a brief conversation with a ward nurse regarding changes in behaviour over the last 7 days, if this information was available. PainChek was completed during rest and post-movement, e.g. moving from bed to chair or repositioning in bed. Researchers sat nearby, not intruding on personal space or observing personal care. Data (apart from PainChek) were collected onto paper forms.

#### Sequence of assessments

Each assessment was completed by two raters (A and B). Rater A collected data on the Abbey Pain scale (at rest), Pain Assessment in Advanced Dementia (PAINAD) and PainChek (at rest and post-movement). Rater B simultaneously but independently collected PainChek data at rest and post-movement and the Symptom Management–End of Life in Dementia (SM-EOLD) scale.

### Study measures

#### PainChek

The automated facial analysis technology identifies facial action units indicative of pain, in real time, using AI-powered algorithms [19, 20]. Pain was first assessed by undertaking a 3-s scan of the participant’s face (Domain One; automatically measuring nine facial features of pain) using an app on an Apple iPad mini-4 (IOS 10). After the AI facial assessment, five other domains are manually scored by the rater on the app: Voice (nine features), Movement (seven features), Behaviour (seven features), Activity (four features) and Body (six features). Each feature is given a score of 1. A final total pain score and severity score are calculated automatically by totalling the six domain scores (maximum score 42), with severity categorised: none (0–6), mild (7–11), moderate (12–15) and severe pain (16–42). Total completion time is 1 minute. PainChek has CE Mark and Therapeutic Goods Administration clearance as a Class 1 Medical Device and regulatory clearance in Singapore (Health Sciences Authority), UK (Medicines and Healthcare products Regulatory Agency) and Canada (Health Canada) [19–23].

#### Pain Assessment in Advanced Dementia

PAINAD is a brief observational pain tool, taking 2 minutes to complete with sensitivity, clinical utility and strong psychometric properties [12], selected because PainChek has not yet been validated against the PAINAD [12]. PAINAD comprises five domains (breathing, negative vocalisations, facial expression, body language and consolability), each scored from 0 to 2 points (maximum 10). A cut-off score of ≥2 indicates the presence of pain [26]. Pain was observed at rest and post-movement.

#### Abbey Pain Scale

The Abbey Pain Scale (APS) evaluates pain intensity by observation. It comprises six domains (vocalisation, facial expression, change in body language, behavioural change, physiological change, physical change), rating each from 0—absent to 3—severe [13]. Maximum score is 18 with severity categorised as none (0–2), mild (3–7), moderate (8–13) and severe (14+). The APS was measured at rest to allow comparison with previous PainChek validation studies.

#### Symptom Management–End of Life in Dementia

SM-EOLD is a reliable and validated observational scale for uncomfortable physical symptoms such as shortness of breath [27], scored 0–45 with higher scores indicating better symptom control.

From hospital notes, we documented sociodemographic details (age, gender, ethnicity, first language, place of residence), medical history—Cumulative Illness Rating Scale-Geriatric (CIRS-G) [28] and dementia severity—Clinical Dementia Rating (CDR) [29].

### Data analysis

Summary measures are presented for participant characteristics as mean and standard deviation (SD) for continuous variables, and frequencies and percentages for categorical variables, based on observed data only, analysed using Stata 17 [30].

#### Reliability

Inter-rater reliability was assessed by classifying PainChek scores into standard cut-offs—none, mild, moderate and severe pain—using Cohen’s kappa, giving a measure of exact agreement within categories. With sparse numbers standard Cohen’s kappa can give misleading results. Therefore, we also calculated Gwet’s alpha coefficient, a paradox-resistant alternative [31]. Interpretation is arbitrary: values ≤0 indicate no agreement; 0.01–0.20, none to slight; 0.21–0.40, fair; 0.41–0.60, moderate; 0.61–0.80, substantial; and 0.81–1.00, almost perfect agreement [32]. Internal consistency between PainChek items was calculated using Cronbach’s alpha at rest and post-movement. Values of Cronbach’s alpha above 0.7 indicate good agreement [33].

#### Validity

We assessed concurrent validity with Pearson’s correlation coefficient (95% confidence intervals, 95% CIs) between PainChek and PAINAD scores at rest and post-movement. To allow comparison with previous PainChek validation studies, we repeated this analysis using PainChek and APS data at rest. Correlation is not a measure of exact agreement, as instruments are based on different scoring mechanisms, but strong correlation indicates that PainChek and PAINAD scores are equivalent up to a scaling factor.

Convergent validity was assessed using Pearson’s correlation coefficient between scores at rest and post-movement on the PainChek and the SM-EOLD. Interpretation of Pearson’s correlation coefficient is arbitrary: 0–0.10 suggests negligible correlation; 0.10–0.39, weak; 0.40–0.69, moderate; 0.70–0.89, strong; and 0.90–1.00, very strong correlation [34].

Discriminant validity assesses whether a measure designed for a particular construct *does not* correlate with a test measuring a different construct. We would expect a difference in pain measurement under the two separate conditions of rest and post-movement. A mixed-effects regression model was used to compare agreement between PainChek and PAINAD (dependent variable) at rest and post-movement with timing as the independent variable. The model considered correlations between repeated measures made on each participant. The *P*-value indicates the influence of timing on the agreement between measures.

### Sensitivity analyses

PainChek was designed for care homes and includes six items requiring a weeks’ previous knowledge of the resident (1—introvert (unsocial) or altered behaviour, 2—inappropriate behaviour, 3—resisting care, 4—prolonged resting, 5—altered sleep–wake cycle, 6—altered routines). This prior information may not be available in hospitals. To investigate whether this affected the psychometric properties of PainChek, we repeated inter-rater reliability and concurrent validity tests, removing the six items that require 7 days’ prior knowledge of the person.

## Results

### Recruitment

**Figure 1:**
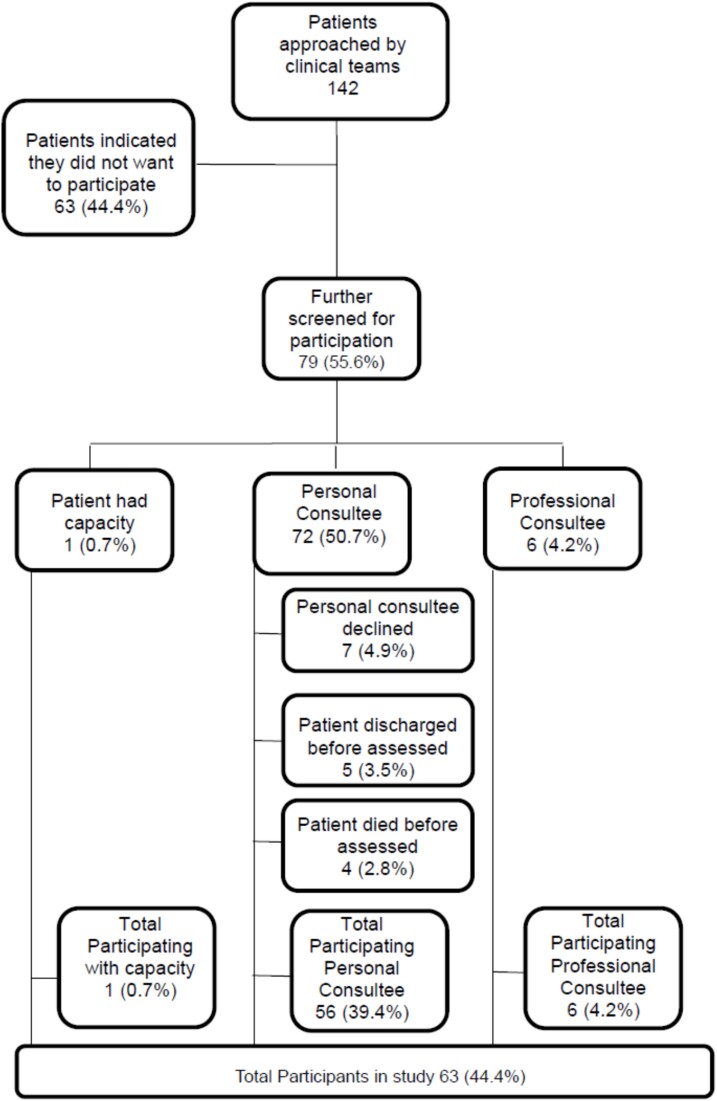
Recruitment flowchart.

The first patient was assessed and recruited on 8 August 2019 at hospital 1. The study was suspended in March 2020 (COVID pandemic and visiting restrictions) and recommenced in July 2022 where we opened site 2. In total, 142 patients and their next-of-kin were approached; 79 consented to meeting a researcher, and 63 were included in the study, the majority of whom were unable to consent for themselves (Fig. 1).

### Cohort characteristics

Mean participant age was 84 years (SD 6.7), 59% were female, the majority were still living in their own homes (69%), white British ethnicity (64%), spoke English as a first language (77%) and had moderate or severe dementia (61%) (Table 1).

**Table 1:** Demographic and clinical characteristics of study participants.

	*N*	Number/mean	%/SD
Age	63	84.0	(6.7)
Gender	63		
Female		37	(58.7)
Male		26	(41.3)
Ethnicity	61		
White British		39	(63.9)
Asian		2	(3.3)
Black		14	(23.0)
Other		6	(9.8)
Language	60		
English		46	(76.7)
Non–English speaking		14	(23.3)
Residence	61		
Home		42	(68.9)
Supported accommodation		3	(4.9)
Residential home		4	(6.6)
Nursing home		10	(16.4)
Mental health ward		2	(3.3)
Consent relationship	63		
Family member		56	(88.9)
Professional consultee		6	(9.5)
Personal consent		1	(1.6)
CDR	62		
0.5 very mild		12	(19.3)
1 mild		12	(19.3)
2 moderate		15	(24.2)
3 severe		23	(37.1)
Comorbidities (CIRS-G total score)	62	12.0	(4.9)
*Musculoskeletal and skin (CIRS-G subscale)*			
0 (no impairment)		21	(33.9)
1		8	(12.9)
2		26	(41.9)
3		5	(8.1)
4		2	(3.2)
5 (extremely severe life threatening)		0	(0.0)

We conducted 216 PainChek assessments; 60 participants were observed by two independent raters at rest and 48 participants observed by two independent raters post-movement. The mean PainChek score (combined for observer 1 and 2) was 3.6 (SD 3.3) at rest and 5.3 (SD 4.8) post-movement. The mean PAINAD score was 1.5 (SD 2.0) at rest and 2.6 (2.8) post-movement, and mean APS score was 2.6 (3.1) (Table 2).

**Table 2:** Pain and discomfort scores in study participants.

	*N*	Number/mean	%/SD
**PainChek**			
Total score at rest (observer 1 and 2)	120	3.6	(3.3)
Total score post-movement (observer 1 and 2)	96	5.3	(4.8)
**PainChek level at rest (observer 1 and 2)**	120		
None		103	(85.8)
Mild		13	(10.8)
Moderate		1	(0.8)
Severe		3	(2.5)
**PainChek level post-movement (observer 1 and 2)**	96		
None		64	(66.7)
Mild		23	(24.0)
Moderate		6	(6.3)
Severe		3	(3.1)
**PAINAD—total score**			
Total score at rest	63	1.5	(2.0)
Total score post-movement	35	2.6	(2.8)
**PAINAD—at rest**	63		
None (0–1)		38	(60.3)
Pain present (≥2)		25	(39.7)
**PAINAD—post-movement**	35		
None (0–1)		17	(48.6)
Pain present (≥2)		18	(51.4)
**Abbey Pain Scale at rest**	63		
Total score		2.6	(3.1)
None (0–2)		36	(57.1)
Mild (3–7)		23	(36.5)
Moderate (8–13)		3	(4.8)
Severe (14+)		1	(1.6)
**Abbey Pain Scale type of pain**	54		
Acute		1	(1.9)
Chronic		38	(70.4)
Acute on chronic		15	(27.8)
**SM-EOLD**	60	30.8	(9.5)

### Reliability

#### Inter-rater reliability

Results suggested substantial agreement between raters at both rest and post-movement using PainChek total scores and categories (Supplementary material—Fig. 1). Inter-rater reliability improved in sensitivity analyses where items requiring prior knowledge of the patient were removed (Table 3).

**Table 3:** Inter-rater reliability of PainChek.

	Rest (*n* = 60)	Movement (*n* = 47)
**Total PainChek score**		
Correlation coefficient (95% CI)	0.714 (0.562 to 0.81)	0.817 (0.692 to 0.894)
ICC (95% CI)	0.690 (0.531 to 0.802)	0.742 (0.581 to 0.847)
**PainChek categories**		
Observed agreement (95% CI)	78.3% (67.6 to 89.1)	70.2% (56.0 to 84.4)
Cohen’s kappa (95% CI)	0.150 (−0.088 to 0.388)	0.420 (0.198 to 0.641)
Gwet’s AC (95% CI)	0.764 (0.638 to 0.889)	0.643 (0.469 to 0.818)
**Sensitivity analyses**		
**Total PainChek score**		
Correlation coefficient (95% CI)	0.779 (0.654 to 0.862)	0.838 (0.726 to 0.907)
ICC (95% CI)	0.776 (0.652 to 0.859)	0.805 (0.676 to 0.886)
**PainChek categories**		
Observed agreement (95% CI)	86.7% (77.8 to 95.5)	72.3% (58.4 to 86.3)
Cohen’s kappa (95% CI)	0.280 (−0.007 to 0.568)	0.326 (0.093 to 0.559)
Gwet’s AC (95% CI)	0.858 (0.759 to 0.957)	0.680 (0.510 to 0.850)

Gwet’s AC, Gwet’s alpha coefficient; ICC, intra-class correlation coefficient

#### Internal consistency

Using the 42 true/false items, Cronbach’s alpha (scale reliability coefficient) at rest was 0.755, showing good internal consistency, and average inter-item covariance was 0.0050. Post-movement, this was 0.833 and average inter-item covariance was 0.011.

### Validity

#### Concurrent validity

We found moderate correlation between PainChek and PAINAD scores at rest and a strong correlation post-movement. [34]. There was a strong correlation between PainChek and the APS at rest. Using standard cut-offs (see methods), we found substantial agreement at rest between categorised (none, mild, moderate and severe) APS [13] and PainChek scores [21] (60.0%: 95% CI 46.8% to 73.2%) (Appendix 1 in Supplementary Data). Removing PainChek items requiring 7 days of previous knowledge of the patient had minimal impact on the concurrent validity of PainChek, compared with PAINAD and APS.

#### Convergent validity

The correlation coefficient between PainChek and SM-EOLD at rest was −0.555 (95% CI −0.726 to −0.318) and the correlation coefficient between PainChek and SM-EOLD post-movement = −0.564 (95% CI −0.733 to −0.331).

#### Discriminant validity

The mixed-effects regression model showed agreement between PainChek and PAINAD, which was significantly influenced by the condition of the assessment with a difference between rest/post-movement [coefficient = 0.858 (95% CI 0.086 to 1.63); *P* < .001] (Table 4).

**Table 4:** Concurrent validity of PainChek.

	PainChek (correlation coefficient, 95% CI)	
**PAINAD**	**Rest (*n* = 60)**	**Movement (*n* = 47)**
Rest	0.528 (0.317 to 0.690)	
Movement		0.787 (0.604 to 0.891)
**Abbey Pain Scale**		
Rest	0.762 (0.630 to 0.851)	
**Sensitivity analyses** ^**a**^		
**PAINAD**		
Rest	0.524 (0.311 to 0.686)	
Movement		0.755 (0.552 to 0.874)
**Abbey Pain Scale**		
Rest	0.748 (0.610 to 0.842)	

^a^Items that required 7 days of previous knowledge of the patient were removed

## Discussion

We explored the psychometric properties of PainChek in people with dementia admitted to hospital. To contextualise our findings, it is important to consider our cohort and setting. Previous validity and reliability studies were set in care homes [22, 23, 35], where residents had more advanced dementia and higher care needs. We recruited people with unplanned admissions to general hospitals, and 69% were still living at home. Compared to previous PainChek studies, our participants were of similar age [22, 23, 35], but more were male and had less severe dementia. We measured the APS at rest in our participants so we could compare pain characteristics of our general hospital cohort against those studied in care homes. The proportion of acute hospital in-patients with mild (36.5%), moderate (4.8%) or severe pain (1.6%) at rest on the APS was higher than in care homes [22], possibly because participants were recruited from hospital wards and more likely to have acute pain.

Overall, PainChek scores in the general hospital showed substantial inter-rater reliability at rest and post-movement, similar to those found in care home residents [21]. Our finding of lower inter-rater reliability at rest is consistent with the literature [35], suggesting that pain measurement is more reliable after movement provocation [36]. Inter-rater agreement on categorial pain scores was substantial and reflected care home cohorts [21]. Internal consistency of PainChek was similar to care home populations [21, 35] and other observational pain tools used in general hospitals [37]. Initial reliability testing of PainChek was in care homes where staff have longer-term knowledge of residents. In hospitals, staff will not have this longitudinal information. In sensitivity analysis, when removing items requiring previous knowledge of the patient, inter-rater reliability of PainChek improved, suggesting that items requiring longitudinal patient knowledge may be less useful in hospitals.

PainChek showed a similar concurrent validity, compared with the APS at rest, to that of care home populations [21, 35]. We would expect good concurrent validity between PainChek and APS as they are based on similar constructs and conceptual foundations [20]. We found the concurrent validity of PainChek against the PAINAD scale was lower at rest than post-movement. There are several possible explanations for this. The PAINAD tool relies on only five behavioural indicators: breathing, negative vocalisation, facial expression, body language and consolability. It has been argued that the PAINAD item of consolability is difficult to interpret and may impact the validity of PAINAD [38, 39]. In addition, the PAINAD ‘breathing’ item tends to be infrequently scored and may lack utility, also reducing validity [12, 40]. Concurrent validity between PainChek and PAINAD was higher post-movement, a common finding in observational pain tools, where movement reveals pre-existing pain or provokes new pain [41]. Removing items requiring previous knowledge of the patient over the prior 7 days had little impact on concurrent validity, possibly because participants were recruited after 48 h in hospital, giving staff time to build knowledge about them.

There are no previous data on the convergent validity of PainChek. We found only moderate convergent validity with the SM-EOLD. Observational pain assessment tools assume the cause of discomfort is pain. Tools such as the SM-EOLD measure a broader spectrum of discomfort. Although pain involves discomfort, discomfort is not invariably the result of pain [42]. We assessed discriminant validity by examining pain scores before and post-movement, finding a significant difference. This suggests that PainChek may be useful in distinguishing between pain and discomfort due to non-pain (non-nociceptive) causes.

### Strengths and limitations

This is the first study to assess the psychometric properties of PainChek in people with dementia admitted to a general hospital. We recruited a relatively large number of participants compared to previous PainChek studies [22, 35]. We did not, however, recruit to target, due to the COVID pandemic which significantly impacted our ability to work in hospitals. Research staff received standardised training in using PainChek. It is possible that there was selection bias as 44% of eligible participants declined to participate. However, we tested the tool in a real-world clinical setting, with a sample representative of people with dementia who have unplanned admissions to medical wards, in terms of age [1, 2, 43], ethnicity [4, 43], place of residence [1, 43] and comorbidity [44]. We recruited participants with severe dementia, who did not have capacity to consent to participation, further ensuring a representative cohort. We included people with milder dementia which may affect the reliability of some findings. However, it is important to consider how a person with mild dementia, who is in an unfamiliar environment, physically unwell and without hearing aids or glasses may find communication more difficult in hospital.

Research staff were blinded to participants’ medical history, pain history and analgesic use, but spoke with the ward nurse about the patients’ behaviour that day. This facilitated independent testing of PainChek but does not reflect how it is used in care homes, where staff know residents, and may be more aware of individual pain behaviours. However, this mirrors the real-world setting of the general hospital ward. Self-report is the gold standard for pain assessment [5], but this was not possible in our population. We chose the APS and PAINAD as reference standards as these are recommended in a range of clinical settings. The automated facial coding system provided by PainChek overcomes lack of prior knowledge about the person’s typical pain reactions which is beneficial in general hospitals.

## Conclusion

PainChek shows potential for use in people with dementia in general hospitals, has construct validity and performs well in comparison to other commonly used clinical pain tools such as the APS and the PAINAD. It needs further optimisation for roll-out into acute care settings. There is a move away from pen and paper–based clinical tools and electronic documentation is more widespread in UK hospitals and global health systems. PainChek would need to be integrated with electronic patient record systems. Importantly, detection of pain is not enough, and appropriate decision support and guidance for pain management needs to be properly implemented [14]. The routine use of electronic pain tools in hospitals would require careful integration into existing workflows and practices [14, 15].

## Supplementary Material

aa-24-1817-File002_afaf027

## Data Availability

The data underlying this article are available from the corresponding author on reasonable request.
