# Supplementary material for: Evaluation of the psychometric properties of PainChek in older general hospital patients with dementia
Source: Age Ageing. 2025 Feb 19;54(2):afaf027. doi: 10.1093/ageing/afaf027 (PMC11836425; doi:10.1093/ageing/afaf027)
Supplement: aa-24-1817-File002_afaf027 [file aa-24-1817-file002_afaf027.docx]

**Appendix 1-Supplementary Data. Agreement on pain assessment at rest between categorized Abbey Pain Scale and PainChek scores**

|  | Abbey Pain Scale at rest (n=60) | | | | |
| --- | --- | --- | --- | --- | --- |
| PainChek  at rest (n=60) | none | mild | moderate | severe | Total |
| none | 32 | 17 | 0 | 0 | 49 |
| mild | 2 | 5 | 2 | 0 | 9 |
| moderate | 0 | 0 | 1 | 1 | 2 |
| severe | 0 | 0 | 0 | 0 | 0 |
| Total | 34 | 22 | 3 | 1 | 60 |

Agreement observed between PainChek and APS. 63.3% (95% CI: 49.9% to 75.4%) at rest

(60.0%: 95% CI: 46.8% to 73.2%)
